# *Grifola frondosa* Polysaccharides Alleviated Cyclophosphamide—Induced Intestinal Injury Based on Microbiota, Metabolite and Immune Axis Modulation

**DOI:** 10.3390/foods14193376

**Published:** 2025-09-29

**Authors:** Jindi Wu, Guilu Chen, Dingfeng Chen, Haoran Zhang, Huirong Lv, Zhengshun Wen

**Affiliations:** 1 School of Food and Pharmacy, Zhejiang Ocean University, Zhoushan 316022, China; wujindi@zjou.edu.cn (J.W.); chenguilu@zjou.edu.cn (G.C.); chendingfeng@zjou.edu.cn (D.C.); 2 Xianghu Laboratory, Hangzhou 311231, China; 2024881084@zwu.edu.cn (H.Z.); lvhuirong1215@163.com (H.L.); 3 College of Biological & Environmental Science, Zhejiang Wanli University, Ningbo 315100, China

**Keywords:** *Grifola frondosa* polysaccharides, intestinal barrier injury, gut microbiota, metabolite

## Abstract

*Grifola frondosa* polysaccharides (GFP), which possess antitumor properties, can counteract intestinal injury induced by cyclophosphamide (CTX). The objective of this research was to evaluate the efficacy of GFP in protecting the intestinal barrier of mice and investigate the mechanisms behind this effect. Using a CTX-induced intestinal barrier injury model, we found that GFP treatment significantly alleviated body weight loss and organ atrophy, while enhancing serum IgG and IgM levels. Histological analysis showed that GFP effectively repaired the intestinal mucosal structure, increased goblet cell numbers, and led to an upregulation in the gene expression of ZO-1, Occludin, and MUC2. GFP modulated cytokine expression, including IFN-γ, IL-4, IL-10, and IL-22. According to 16S rDNA sequencing results, GFP enhanced the abundance of *unclassified*_*Muribaculaceae* while reducing the prevalence of *Escherichia_Shigella*. Furthermore, GFP elevated the concentrations of several metabolites, including SCFAs and pyridoxal, which are closely related to intestinal barrier protection and mucosal immunity. Overall, this study demonstrated that GFP has strong potential as an immune-enhancing adjuvant and may represent a promising intervention strategy to mitigate chemotherapy-induced intestinal injury.

## 1. Introduction

*Grifola frondosa* is an edible fungus with important medicinal value, containing key bioactive constituents such as polysaccharides, triterpenoids, phenols, and flavonoids [1]. In particular, polysaccharides from *Grifola frondose* (GFP) are abundant and have attracted significant research interest owing to their broad pharmacological activities. GFP has demonstrated notable immunomodulatory [2], antioxidant [3], anti-inflammatory [4], and antitumor properties [5]. A starch-like GFP exerts its immunomodulatory function by promoting lymphocyte proliferation and activating the MAPK signaling pathway in CTX-induced immunosuppressed mice [6]. Oral administration of low-molecular-weight GFP has been shown to protect immune organs and induce apoptosis in tumor cells in H22 tumor-bearing mice [7]. However, the protective mechanism of GFP in chemotherapeutic drug-induced intestinal injury remains unclear.

The intestine, the body’s largest peripheral immune organ, is not only central to digestion and absorption but also serves as a key barrier to maintain overall health [8,9]. The mucosal barrier is primarily constituted by highly O-glycosylated mucins and functions to maintain homeostasis in the intestinal epithelium (e.g., MUC2), which not only ensures that the epithelium is in a near-sterile environment but also prevents the invasion of pathogens, removes toxins, and lubricates the intestinal tract [10,11,12]. Lymphocytes and their surrounding lymphoid tissues form the intestinal mucosal immune system. This system is pivotal in the host’s defense against pathogenic invasion and in the preservation of a balanced intestinal flora, largely through the action of effector molecules such as secretory immunoglobulins. Abnormalities in intestinal immunoregulatory function cause pathologic changes in the gut and may affect the systemic immune homeostasis of the host. The intestinal micro-environment is a dynamic ecosystem formed by intestinal flora and their metabolites through complex interactions. The intestine harbors a vast and diverse community of microorganisms that decompose complex carbohydrates into SCFAs. These SCFAs strengthen tight junctions between epithelial cells, reducing “leaky gut” syndrome [13] and stimulate epithelial mucus secretion, thereby enhancing the mucus barrier to block pathogen invasion [14]. Moreover, microbiota-derived metabolites can directly modulate immune cells, exerting potent immunoregulatory effects [15,16].

Cyclophosphamide (CTX) is a clinical alkylating chemotherapeutic agent that often triggers intestinal barrier function damage during the treatment of tumors and autoimmune diseases [17]. The drug causes significant damage to the small intestine through mechanisms such as disrupting intestinal mucosal integrity, increasing intestinal permeability, and disturbing microbiota homeostasis [18]. Polysaccharides from both *Longan pulp* and *Rehmannia glutinosa* attenuate CTX-induced injury to the intestinal barrier by enhancing the production of MUC2 and tight junction proteins [19,20]. In addition, GFP exhibited a protective effect against CTX-induced intestinal injury.

Therefore, the present work was designed to evaluate the protective influence of GFP on the intestinal barrier following injury induced by CTX, with specific emphasis on its role in barrier restoration mediated by the gut microbiota–metabolite–immune network. These findings may support developing GFP into a polysaccharide-based adjuvant for chemotherapy and immunotherapy.

## 2. Materials and Methods

### 2.1. Materials

CTX was obtained from Sigma-Aldrich (St. Louis, MO, USA). The Mouse Diamine Oxidase (DAO) ELISA Assay Kit was acquired from Zhaorui Biotechnology Co., Ltd. (Shanghai, China), and the Lipopolysaccharide/Endotoxin (LPS) ELISA Kit was procured from Ximei Chemical Co., Ltd. (Shanghai, China). Enzyme-linked immunosorbent assay kits of immunoglobulin G (IgG) and immunoglobulin M (IgM) were purchased from Winter Song Boye Biotechnology Co., Ltd. (Beijing, China). FastPure Cell/Tissue Total RNA Isolation Kit V2, HiScript Ⅱ Q RT SuperMix for qPCR (+DNA wiper), ChamQ Universal SYBR qPCR Master Mix were all purchased from Vazyme Biotech Co., Ltd. (Nanjing, China). The short-chain fatty acid standards were purchased from Merck (Shanghai, China).

### 2.2. Extraction of Polysaccharides from Grifola frondosa

GFP was extracted using a method slightly modified from previous research [21]. In brief, the dried *Grifola frondosa* was ground into powder and accurately weighed. Distilled water was added at a ratio of 1:15 (*w*/*v*) and extracted under boiling water bath conditions for 2 h. Filtrate was centrifuged at 12,000× *g* for 10 min, and the supernatant was collected. The residue was extracted twice under the above conditions, and the supernatant was combined 3 times; the combined supernatant was concentrated to 1/4 of the original volume by rotary evaporator; and 4 times the volume of anhydrous ethanol was added slowly into the concentrated solution, which was allowed to stand at 4 °C overnight, and the precipitate was centrifuged and collected. The solution was treated with Sevag reagent to remove the protein, and the deproteinized solution was packed into a dialysis bag with a molecular weight cut-off of 1000 Da, and dialyzed by flowing distilled water for 48 h. The dialysate was freeze-dried at −60 °C for 72 h, and then the *Grifola frondosa* polysaccharides were obtained. The extraction yield was about 6.4%. The structural characterization of GFP is shown in Figure S1.

### 2.3. Animal and Experimental Design

**Figure 1:**
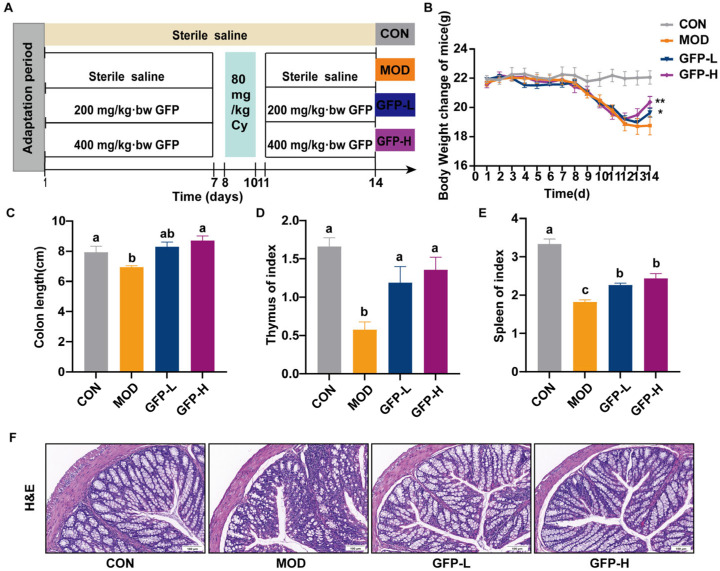
Effect of GFP on CTX-induced intestinal barrier injury in mice. (**A**) Diagram of experimental schedule. (**B**) The change in body weight. (**C**) Colon length. (**D**) Spleen index. (**E**) Thymus index. (**F**) Representative image of H&E-stained pathological section of colon tissue (20×, scale bar: 100 μm) (each group *n* = 3). Means with different letters were significantly different (*p* < 0.05). * *p* < 0.05, ** *p* < 0.01.

Six-week-old female specific pathogen-free (SPF) BALB/c mice (20–22 g) were purchased from Vital River Laboratory Animal Technology Co., Ltd. (Pinghu, China). All animal experiments complied with ARRIVE guidelines and were approved by the Animal Ethics Committee of Zhejiang Academy of Agricultural Sciences (Certificate: number: 25ZALAS45). The animals were housed under controlled conditions with a humidity of (50 ± 5%), temperature of (22 ± 2 °C), a 12 h light/dark cycle, and allowed to feed and drink freely. The experimental design is illustrated in Figure 1A: After one week of acclimatization, the mice were randomly divided into 4 groups (8 mice/group), including the CON group, MOD group, GFP-L group, and GFP-H group. From day 1 to day 7, the CON and MOD groups received saline by gavage, whereas the GFP-L and GFP-H groups were administered low-dose (200 mg/kg BW) and high-dose (400 mg/kg BW), respectively. From day 8 to day 10, except for the CON group, which received intraperitoneal injections of saline, the MOD group, GFP-L group, and GFP-H group were intraperitoneally injected with CTX (80 mg/kg BW) for three consecutive days to establish the model. During day 11 to day 14, saline was administered to the CON and MOD groups by gavage, with the GFP-L and GFP-H groups receiving 200 and 400 mg/kg BW GFP, respectively. At the end of the experiment, blood was taken from the orbits, and samples of spleen, thymus, colon tissue, and cecal contents were rapidly frozen at −80 °C for later use.

### 2.4. Evaluation of Spleen and Thymus Indices

A sterilized scalpel and forceps were used to isolate the spleen and thymus, followed by removal of adherent fat and surface connective tissue. The organs were promptly rinsed in sterile PBS to eliminate bloodstains, then blotted dry on filter paper. Weigh immediately and record. The spleen and thymus indices were calculated as follows: the spleen or thymus indices were expressed as spleen or thymus weight/body weight × 100.

### 2.5. Measurement of Relevant Indicators in Serum

Blood was collected from the orbital sinus and left to stand for 1 h at room temperature, then centrifuged at 3000× *g* for 15 min at 4 °C to obtain serum. Following the manufacturer’s instructions, the levels of immunoglobulins (IgM, IgG) and the contents of diamine oxidase (DAO) and lipopolysaccharide (LPS) in the serum were determined using ELISA kits.

### 2.6. Histopathological Analysis

A section of colon tissue without fecal residue was taken and fixed by 4% polyformaldehyde for 24 h, then sequentially subjected to gradient ethanol dehydration, xylene hyalinization, and paraffin embedding. The colonic tissues, embedded in paraffin, were subjected to Hematoxylin and eosin (H&E) or Alcian blue-periodic acid Schiff (AB-PAS) staining for histological examination of pathological changes and mucus production.

### 2.7. qRT-PCR Analysis

The FastPure Cell/Tissue Total RNA Isolation Kit V2 was used to extract total RNA from colon samples. Total RNA was converted into cDNA employing the HiScript Ⅱ Q RT SuperMix for qPCR kit. Quantitative real-time PCR (qPCR) was conducted using the QuantStudioTM Design (Software v1.X) & Analysis Software Real-Time System. The 10 μL reaction volume consisted of 5 μL 2 × ChamQ Universal SYBR qPCR Master Mix, 0.2 μL of forward primer, 0.2 μL of reverse primer, 2 μL of cDNA template, and 2.6 μL of ddH_2_O, which was used for quantitative real-time PCR detection. The primer sequences used in this study are listed in Table 1.

### 2.8. Immunofluorescence Analysis

Immunofluorescence (IF) was used to observe the expression levels of ZO-1, Occludin, and MUC2 in colon tissues. The sections of each group were dewaxed, rehydrated, rinsed, and then placed in EDTA antigen repair buffer (pH 8.0) for repairing. After adding autofluorescence agent for 5 min and rinsing with PBS for 10 min, the sections were incubated with BSA for 30 min, washed off the sealing solution, incubated with diluted primary antibodies (e.g., anti-ZO-1, Occludin and MUC2) overnight at 4 °C, added with the secondary antibody of the corresponding genus to cover the tissues, and then incubated in a protected environment for 50 min. The sections were sealed with the nuclei of the cells by adding a drop of DAPI and then cured. Sections were examined by fluorescence microscopy, and representative images were captured.

### 2.9. Determination of SCFAs Content in Cecal Contents

The levels of SCFAs were analyzed by referencing previously established research methodologies [22]. A total of 50 mg of cecal content was homogenized in 500 μL of saline, then 20 μL of sulfuric acid, followed by 800 μL of ether, was added to extract the SCFAs and was mixed well, and then the supernatant was centrifuged at 12,000× *g* for 15 min to collect the supernatant. The supernatant was added to a 2 mL EP tube containing 0.25 g of anhydrous sodium sulfate, and then the supernatant was centrifuged under the same conditions again. The supernatant was added to a 2 mL EP tube containing 0.25 g of anhydrous sodium sulfate and centrifuged again under the same conditions to remove residual water. The supernatant was filtered through a 0.22 μm organic filter membrane for GC-MS analysis.

### 2.10. 16S rDNA Sequencing

Total microbial genomic DNA was extracted from fecal samples using the E.Z.N.A.^®^ Stool DNA Kit DNA Extraction Kit (Norcross, GA, USA). The extracted DNA served as the template for amplifying the V3–V4 hypervariable region of the 16S rRNA gene with primers 338F and 806R. After PCR purification, PE libraries were constructed using the TruSeqTM DNA Sample Prep Kit (San Diego, CA, USA) and sequenced using the Illumina platform (San Diego, CA, USA). Species composition analysis, sample comparison analysis, and LEfSe were used to observe the changes in microbial communities.

### 2.11. Untargeted Metabolomics Analysis of Fecal

An untargeted metabolomics approach was used to determine the changes in metabolites in mouse feces after GFP intervention. The method was as follows: an appropriate amount of colon contents was taken, methanol and water (total volume of 400 µL) were added at a volume ratio of 4:1; colon contents were thus broken at low temperature, followed by being vortexed and mixed well, and then were extracted by ultrasonication on ice. The samples were allowed to stand for 30 min and centrifuged at 13,000× *g* for 10 min, and after centrifugation, the supernatant was taken and transferred to the injection vials for LC-MS analysis. Raw data were imported into Progenesis QI (Waters Corporation, Milford, MA, USA) for calibration and analyzed by Mass spectrometry and MSMS mass spectrometry data, Metlin (http://metlin.scripps.edu, accessed on 6 November 2024), Human Metabolome Database (HMDB; https://hmdb.ca/metabolites, accessed on 6 November 2024), and Kyoto Encyclopedia of Genes and Genomes (KEGG; https://www.genome.jp/kegg/pathway.html, accessed on 8 November 2024) databases to complete the metabolite analysis.

### 2.12. Statistical Analysis

Routine data analysis was performed using GraphPad Prism software (version 9.5; GraphPad Software, San Diego, CA, USA), and differences between multiple groups were analyzed by one-way analysis of variance ANOVA test, with results expressed as mean ± SEM. *p* < 0.05 was considered statistically significant. Significant differences between data are indicated by different letters in the Figures.

## 3. Results

### 3.1. GFP Alleviated Symptoms of Intestinal Barrier Injury

To investigate the immunomodulatory activity of GFP, a CTX-induced intestinal barrier injury model was used. As shown in Figure 1B, all groups exhibited steady body weight gain during the first 7 days of feeding (prior to CTX injection). From day 8 onwards, the body weight of all groups except the CON group declined due to CTX administration, indicating systemic damage. Compared with the MOD group, both GFP-treated groups exhibited slower weight loss and subsequent recovery, with the GFP-H group showing the most pronounced improvement. This suggests a dose-dependent protective effect of GFP against CTX-induced damage. Colon length was markedly reduced in the MOD group, whereas GFP supplementation markedly restored colon length (Figure 1C). As important immune organs, the spleen and thymus are sensitive indicators of immune function. CTX significantly reduced the spleen index in the MOD group, while GFP—especially at the high dose—significantly increased both spleen and thymus indices (Figure 1D,E), suggesting attenuation of immune organ atrophy. Crypt morphology was severely disrupted in the MOD group but was largely restored in GFP-treated mice, approaching normal architecture (Figure 1F). These findings indicate that GFP effectively mitigates CTX-induced intestinal injury.

### 3.2. GFP Increased Immunoglobulin Levels and Cytokine mRNA Expression

**Figure 2:**
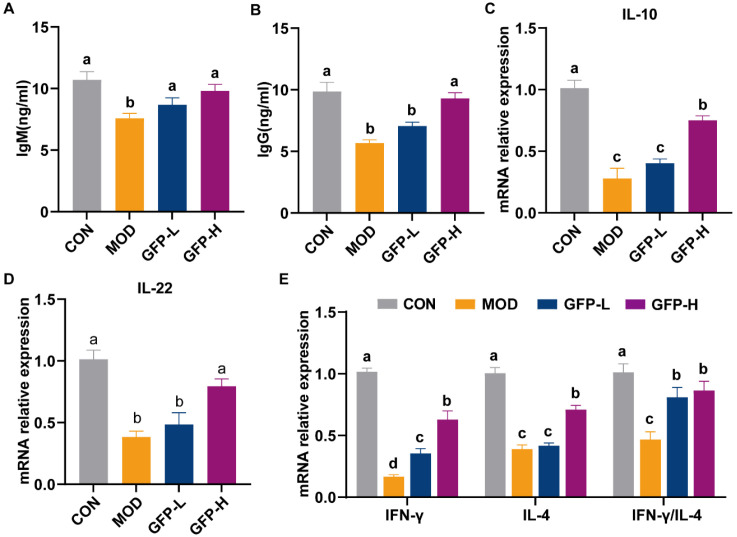
Effect of GFP on mucosal immunity in mice with CTX-induced intestinal barrier injury: Concentration of immunoglobulin (**A**) IgG, (**B**) IgM in serum. Effects of GFP on mRNA expression of (**C**) IL-10, (**D**) IL-22, (**E**) IFN-γ, IL-4, IFN-γ/IL-4. Means with different letters were significantly different (*p* < 0.05).

Immunoglobulins are essential for humoral immunity, mediating pathogen recognition, binding, and activation of immune responses. GFP supplementation significantly increased serum IgG and IgM levels in immunosuppressed mice compared with the MOD group (Figure 2A,B). CTX reduced IL-10 mRNA expression in colonic tissues, whereas GFP significantly restored it (Figure 2C). IL-22, secreted mainly by Th17 and NK cells, supports intestinal barrier homeostasis by promoting epithelial proliferation, differentiation, and antimicrobial peptide secretion. Its expression was downregulated in the MOD group. However, GFP administration restored it in a dose-dependent fashion (Figure 2D). To further assess immune modulation, we examined IFN-γ (Th1-associated) and IL-4 (Th2-associated) expression. CTX significantly suppressed cytokine expression, indicating impaired immune responses (Figure 2E).

### 3.3. GFP Improved Intestinal Barrier Function

**Figure 3:**
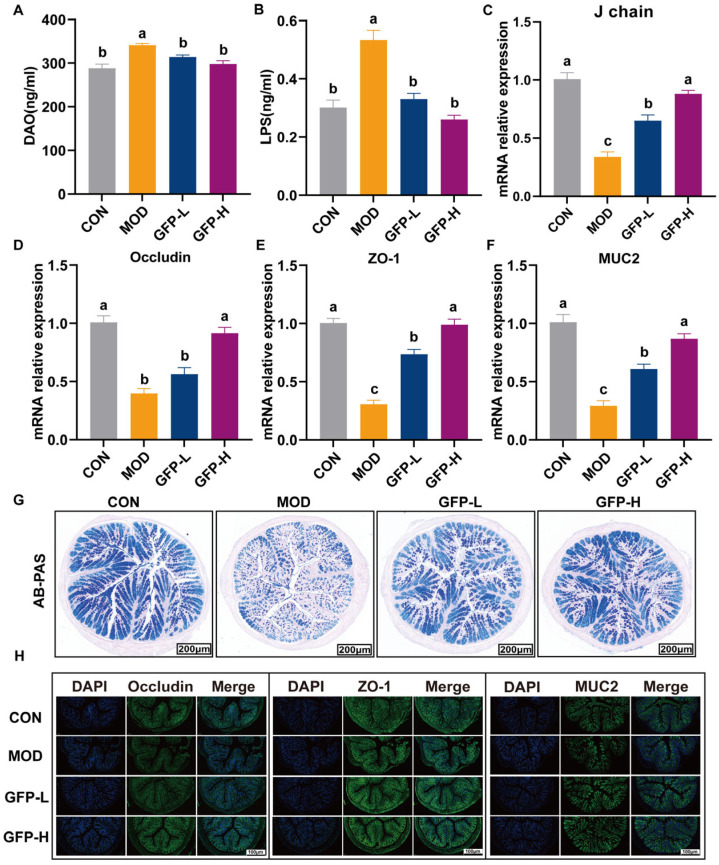
Effect of GFP on intestinal barrier integrity in mice with CTX-induced intestinal barrier injury: (**A**,**B**) The content of DAO, LPS in the serum. The mRNA relative expression level of (**C**) J chain, (**D**) ZO-1, (**E**) Occludin, and (**F**) MUC2 in the colon. (**G**) Representative image of AB-PAS-stained section of colon tissue (7×, Scale bar = 200 μm). (**H**) Representative images of immunofluorescence of ZO-1, Occludin, and MUC2 (12×, scale bar = 100 μm). Different letters were significantly different (*p* < 0.05).

CTX-induced cytotoxicity damages intestinal epithelial cells, leading to increased serum DAO and LPS levels. GFP supplementation significantly reduced DAO and LPS concentrations, with greater efficacy observed in the GFP-H group (Figure 3A,B). Secretory IgA (sIgA) consists mainly of two IgA molecules, a J chain and a secretory component molecule. CTX significantly decreased the J chain and polymeric immunoglobulin receptor (pIgR) expression, whereas GFP treatment restored their levels (Figure 3C and Figure S2). Figure 3D–F shows that CTX significantly reduced their colonic mRNA expression, while GFP supplementation markedly upregulated all three. AB-PAS staining revealed a reduction in goblet cell numbers in the MOD group, which was reversed by GFP treatment (Figure 3G). Immunofluorescence analysis confirmed that CTX reduced the fluorescence intensity of ZO-1, Occludin, and MUC2, whereas GFP restored it (Figure 3H), consistent with gene expression results. These findings indicate that GFP enhances both the mechanical and mucus barriers to protect against CTX-induced intestinal injury.

### 3.4. GFP Restored Gut Microbiota Diversity and Composition

**Figure 4:**
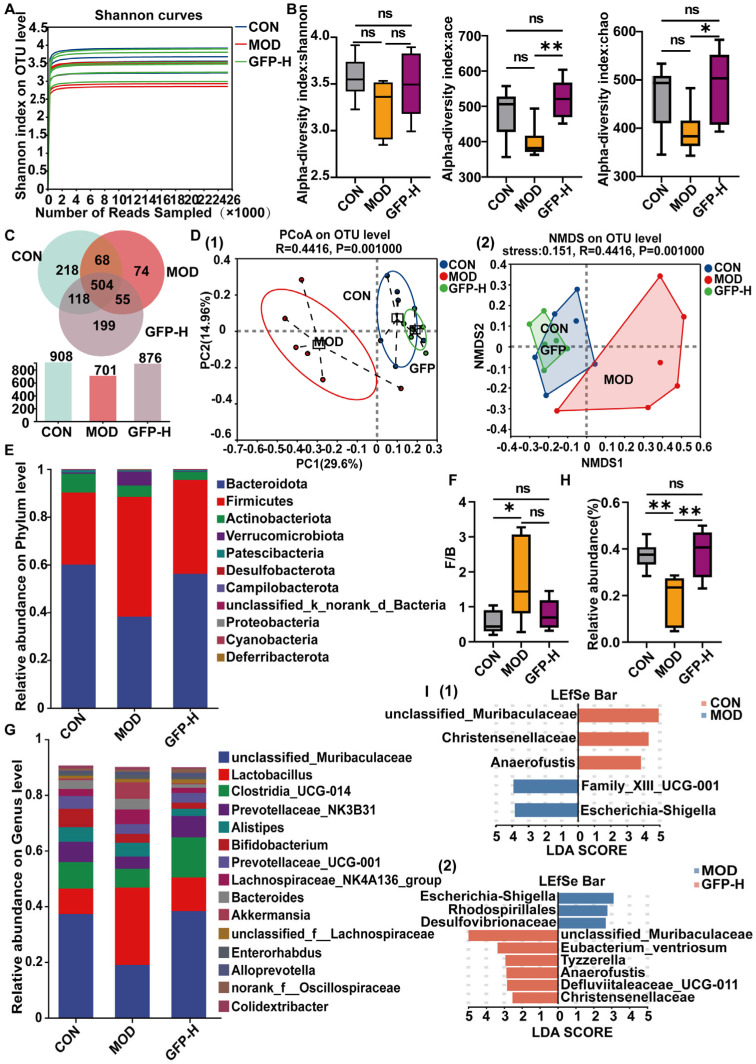
The effects of GFP-H on the gut microbiota in mice with CTX-induced intestinal barrier injury: (**A**) Rarefaction curves. (**B**) α-diversity indices. (**C**) Venn diagram of OTUs. (**D**) (1) Principal co-ordinate analysis (PCoA) of gut microbiota based on bray_curtis distance. (2) Non-metric multidimensional scaling (NMDS) of gut microbiota based on bray_curtis distance. (**E**) Relative abundance of gut microbiota at the phylum level. (**F**) F/B. Note: F/B means Firmicutes/Bacteroidota. (**G**) Relative abundance of bacteria in the top 15 species abundances at the genus level. (**H**) The relative abundances of *unclassified*_*Muribaculaceae*. (**I**) LEfSe analysis (LDA score > 2, *p* < 0.05) (1) CON vs. MOD, (2) MOD vs. GFP-H. * *p* < 0.05, ** *p* < 0.01.

Given that GFP-H produced the strongest protective effects, it was selected for 16S rDNA sequencing. Rarefaction curves indicated adequate sequencing depth (Figure 4A). As shown in Figure 4B, compared with the CON group, CTX decreased the Shannon index, ace index, and chao index, and these indices were significantly increased after supplementation with GFP-H. The ace index and chao index showed significant differences (*p* < 0.05). The Venn diagram showed that the GFP-H group had more operational taxonomic units (OTUs) than the MOD group, approaching CON group levels (Figure 4C). PCoA and NMDS demonstrated distinct clustering among the three groups, suggesting that GFP-H modulated CTX-induced microbial shifts (Figure 4D). At the phylum level, Firmicutes, Bacteroidota, and Actinobacteria were the three dominant phyla, together accounting for over 90% of all sequences (Figure 4E). Relative to the CON group, the Firmicutes abundance was elevated, while that of Bacteroidota was reduced. And the F/B was increased in the MOD group, after supplementation with GFP-H, it recovered to a level comparable to the CON group (Figure 4F). At the genus level, the results show that *unclassified*_*Muribaculaceae*, *Lactobacillus*, *Clostridia*_UCG-014, and *Prevotellaceae*_NK3B31 emerged as the four predominant genera. CTX reduced *unclassified*_*Muribaculaceae*, *Clostridia*_UCG-014, and *Prevotellaceae*_NK3B31 (Figure 4G), while increasing Lactobacillus abundance. Supplementation of GFP-H mitigated the effects of CTX, restored the abundance of *unclassified*_*Muribaculaceae*, *Clostridia*_UCG-014, and *Prevotellaceae*_NK3B31, and reduced the relative abundance of *Lactobacillus* (Figure 4H), returning to normal levels. This suggested that GFP-H alters CTX-induced changes in gut microbiota. LEfSe analysis revealed that the MOD group was enriched in pathogenic taxa such as *Escherichia_Shigella*, *Rhodospirillales*, and *Desulfovibrionaceae*, whereas GFP-H increased beneficial bacteria, including *unclassified*_*Muribaculaceae*, *Christensenellaceae*, and *Eubacterium ventriosum* (Figure 4I).

### 3.5. GFP Modulated Gut Microbiota Metabolism

**Figure 5:**
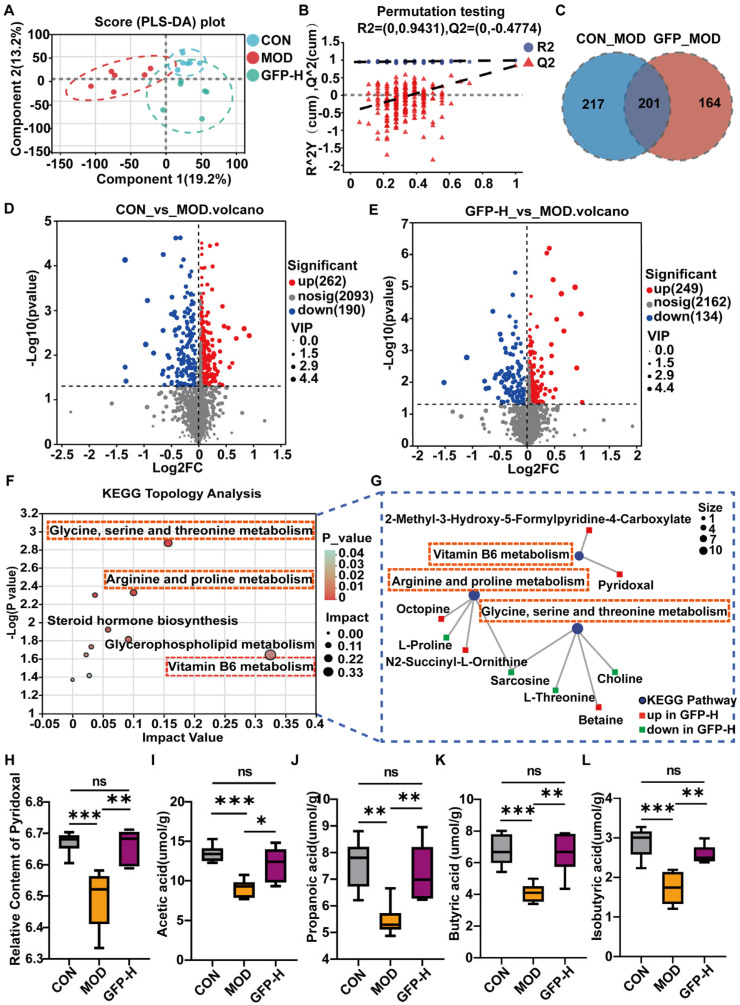
Effects of GFP on the colon metabolome of CTX-induced intestinal barrier injury: (**A**) PLS-DA plots of metabolites among CON, MOD, and GFP-H groups. (**B**) The PLS-DA permutation test. (**C**) Venn diagram of differential metabolites of the comparison groups CON vs. MOD and GFP vs. MOD. (**D**) Volcano plots of metabolites between CON and MOD groups. (**E**) Volcano plots of metabolites between GFP-H versus MOD groups. (**F**) KEGG Topology Analysis. (**G**) Related differentially metabolites in the enriched pathways. (**H**) The relative content of pyridoxal. The content of (**I**) acetic acid, (**J**) propanoic acid, (**K**) butyric acid, (**L**) isobutyric acid. * *p* < 0.05, ** *p* < 0.01, *** *p* < 0.001.

GFP intervention altered metabolites associated with gut microbiota (Figure 5A). The validation of the PLS-DA model indicated that the value of R^2^ exceeded that of Q^2^, indicating good model fit and strong predictability, making it suitable for subsequent data analysis (Figure 5B). The Venn diagram shows 201 identical differential metabolites between CON vs. MOD groups and MOD vs. GFP groups (Figure 5C). As shown in Figure 5D,E, compared with the CON group, the MOD group exhibited a decrease in 190 metabolites and an increase in 262 metabolites. Furthermore, GFP-H treatment modulated 383 metabolites, with 249 being upregulated and 134 downregulated. These metabolites were primarily organic acids and derivatives, lipid and lipid-like molecules, and organic heterocyclic compounds (Figure S3). Pathway analysis identified three significantly affected pathways: vitamin B6 metabolism, glycine, serine, and threonine metabolism, arginine and proline metabolism (Figure 5F). To explore specific metabolites in detail, the levels of key metabolites in the three pathways were analyzed (Figure 5G). Notably, GFP-H increased pyridoxal (vitamin B6 metabolite) (Figure 5H) and modulated several amino acid-related metabolites, including betaine, choline, L-threonine, sarcosine, octopine, and N2-succinyl-L-ornithine.

### 3.6. GFP Increased the Production of SCFAs

CTX reduced SCFA levels, including acetic, propionic, isobutyric, and butyric acids. GFP-H significantly restored these SCFAs (Figure 5I–L), suggesting enhanced microbial fermentation activity.

### 3.7. Correlation Analysis

**Figure 6:**
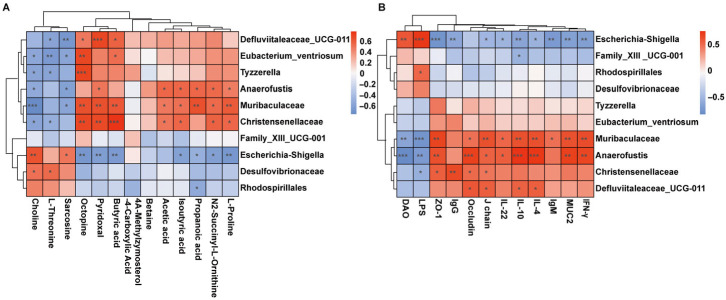
Metabolomic analysis of intestinal microorganisms in mice and the therapeutic effect of GFP-H: (**A**) Correlation of enriched microbiota with key metabolites. (**B**) Association between enriched microbiota and relevant indices. Red indicates a positive correlation, whereas blue indicates a negative correlation. * *p* < 0.05, ** *p* < 0.01, *** *p* < 0.001.

Spearman correlation analysis revealed that choline, L-threonine, and sarcosine were positively associated with *Escherichia_Shigella*, whereas pyridoxal, octopine, SCFAs, and N2-succinyl-L-ornithine correlated positively with beneficial taxa such as *unclassified_Muribaculaceae*, *Anaerofustis*, and *Christensenellaceae* (Figure 6A). Additionally, *Escherichia_Shigella* abundance was positively correlated with serum DAO and LPS levels, indicating a role in promoting intestinal permeability and inflammation. Conversely, *unclassified_Muribaculaceae* and *Anaerofustis* correlated positively with anti-inflammatory cytokine and barrier-associated markers, suggesting a role in immune homeostasis (Figure 6B). Collectively, these results indicate that GFP modulates gut microbiota composition and metabolism, enhancing beneficial bacteria and their metabolites, which in turn contribute to improved immune function and intestinal barrier integrity in CTX-treated mice.

## 4. Discussion

CTX is a commonly used alkylating chemotherapeutic agent, but its therapeutic effects come with side effects such as systemic immunosuppression and intestinal barrier damage [23,24]. Previous research has demonstrated that polysaccharides can effectively regulate the immune barrier and repair the CTX-induced intestinal injury [25]. *Astragalus* polysaccharides enhance the intestinal barrier by modulating the structure of the gut microbiota and increasing the abundance of SCFA-producing microbiota in mice with colitis to increase SCFA levels [26]. *Cordyceps* polysaccharides ameliorate CTX-induced intestinal mucosal immunosuppression and gut dysbiosis in mice [23]. According to established literature, dietary polysaccharides are utilized by gut microbiota through fermentation and metabolic processes, yielding bioactive SCFAs and other metabolic byproducts. These derivatives contribute significantly to the preservation of host immune homeostasis by regulating microbial community structure and reinforcing intestinal barrier integrity [27]. However, the constitutive relationship between polysaccharides and intestinal microbiota and metabolism, along with their protective mechanisms against chemotherapy-induced intestinal injury, remains incompletely elucidated. Therefore, the current research was designed to explore the therapeutic potential of GFP and elucidate its associated molecular mechanisms.

As core components of mucosal immunoglobulins, IgG and IgM secreted by B lymphocytes perform differential functions in the intestinal immune barrier. Serum IgM and IgG levels were reduced in the MOD group relative to the CON group, while GFP treatment resulted in restoration (Figure 2A,B). T helper (Th) cells (Th1/Th2 subsets) maintain immune balance; disruption promotes autoimmune disorders, allergies, and chronic inflammation [28]. IFN-γ and IL-4 are key regulatory cytokines, with their ratio (IFN-γ/IL-4) indicating Th1/Th2 balance. GFP (400 mg/kg BW) significantly increased the IFN-γ/IL-4 ratio (Figure 2E), attenuating CTX-induced mucosal injury by promoting T-cell-mediated immunity and restoring Th1/Th2 homeostasis—consistent with reports on low-molecular-weight *Glycyrrhiza* polysaccharides [29]. The data demonstrated that GFP supplementation improved gut mucosal immunity.

The intestinal barrier is an important defense system for maintaining intestinal homeostasis and body health. DAO is a key indicator of intestinal barrier integrity and reflects mucosal damage. It acts by blocking the translocation of intestinal endotoxins such as LPS into the bloodstream, thus mitigating systemic inflammatory responses [30]. Intestinal barrier damage increases permeability, elevating serum DAO and LPS levels. In this study, the levels of DAO and LPS were elevated in the MOD group and significantly decreased in the serum after GFP treatment (Figure 3A,B). Antigenic stimulations activate gut-associated lymphoid tissue (GALT), inducing differentiation of IgA^+^ plasma cells in the lamina propria and upregulating IgA secretion to form IgA dimers via J chain covalent linkage, which subsequently bind to the poly-immunoglobulin receptor (pIgR) into the epithelial cells to form sIgA in the intestinal lumen [31]. SIgA enhances the mechanical stability of the mucus layer and prevents penetration of pathogens [32]. In this study, we measured the mRNA expression of J chain and pIgR. We found that GFP at 400 mg/kg BW significantly promoted the expression of J chain and pIgR compared with MOD (Figure 3C). Tight junction (TJ) proteins and mucins are important biomarkers that determine intestinal mucosal barrier function and intestinal integrity [33]. In this study, CTX decreased the expression of ZO-1, Occludin, and MUC2, the levels of which were significantly increased after GFP intervention. At the same time, immunofluorescence also showed consistent results.

The gut microbiota constitutes a complex and functionally diverse microecosystem that participates in regulating intestinal mucosal immune responses through multiple mechanisms, playing a pivotal role in maintaining host immune homeostasis [34]. CTX disrupted microbiota composition, while GFP restored balance by promoting beneficial bacteria and suppressing pathogens. Post-CTX modeling, Bacteroidetes abundance decreased significantly, while Firmicutes and the Firmicutes/Bacteroidota (F/B) ratio increased (Figure 4F). As Bacteroidetes degrade carbohydrates and maintain immune balance, an elevated F/B ratio may trigger inflammatory bowel disease (IBD) [35], obesity [36], and various immune disorders [37]. GFP intervention lowered the Firmicutes/Bacteroidota (F/B) ratio and elevated Bacteroidota levels, ameliorating intestinal immune damage. Genus-level analysis showed that CTX induced a significant increase in the abundance of *Lactobacillus* in mice, and GFP treatment effectively reduced the abundance of *Lactobacillus*, which is consistent with Han et al. [38]. The *Muribaculaceae* family degrades complex polysaccharides and produces SCFAs. These SCFAs significantly contribute to the formation of the intestinal mucus layer and enhance epithelial barrier function [39,40]. GFP administration led to a marked increase in the abundance of *unclassified*_*Muribaculaceae*.

The dynamic interplay between gut microbiota and the host immune system critically depends on microbial metabolites. SCFAs are functional metabolites produced by beneficial intestinal bacteria. They not only provide metabolic energy for intestinal epithelial cells but also act as signaling molecules to regulate the function of intestinal barriers [41,42]. A rise in Bacteroidetes abundance—a major source of SCFAs—promotes subsequent elevation in SCFA levels. Acetic acid strengthens mucosal immune defense by activating B cells and promoting IgA production [43]. Propionate demonstrates significant anti-inflammatory and immunoregulatory properties while promoting intestinal barrier repair [44]. Butyrate, as the primary energy source for enterocytes, modulates immune cell metabolism and supports epithelial cell proliferation [45]. In this study, GFP intervention promoted the growth of acetic, propionic, and butyric acid levels (Figure 5I–L). Following GFP intervention, significant alterations were observed in vitamin B6 metabolism, glycine–serine–threonine metabolism, and arginine–proline metabolic pathways. Vitamin B6 deficiency impairs both cellular and humoral immunity, leading to reduced immune competence [46,47]. As the active form of vitamin B6 metabolism, pyridoxal plays essential roles in amino acid metabolism and neurotransmitter synthesis. Its deficiency may impair lymphocyte proliferation, reduce antibody production, and cause abnormal cytokine secretion, consequently compromising the host’s immune response [48].

Correlation analysis showed a significant positive association between *unclassified*_*Muribaculaceae* and SCFAs, consistent with their known synergistic effects in maintaining intestinal barrier function. In contrast, *Escherichia_Shigella* was negatively correlated with propionic acid, butyric acid, and pyridoxal, which suggests the existence of a potential metabolic antagonism. This phenomenon requires further study to elucidate its pathogenic significance. In conclusion, the reparative effect of GFP on CTX-induced intestinal injury in mice is strongly associated with the profile of gut microbial communities and their metabolic products.

## 5. Conclusions

This study demonstrates that GFP effectively mitigates CTX-induced intestinal injury through multiple mechanisms: (1) enhancing the defense capacity of the intestinal immune system by increasing cytokine secretion and upregulating mRNA expression of immune-related pathways; (2) improving intestinal barrier function to reduce pathogen invasion, achieved by augmenting tight junction protein expression and mucin secretion; and (3) restoring intestinal microecological homeostasis by increasing gut microbiota diversity and promoting the production of beneficial metabolites in mice. Collectively, these findings indicate that GFP restores intestinal barrier integrity, modulates gut microbiota composition, and corrects CTX-induced metabolic disturbances. Based on these results, GFP demonstrates potential as a functional dietary component aimed at modulating the gut microbiota and represents a potential adjuvant therapeutic agent for immune-related diseases. However, this study has only provided a preliminary characterization of GFP’s structure. Its fine structure requires further elucidation. Additionally, the specific mechanisms of action and application potential need to be systematically validated through cellular experiments and clinical trials.

## Figures and Tables

**Table 1:** Mouse-gene-specific primer sequences used in RT-qPCR.

Gene	Froward Primer	Reverse Primer
*β-actin*	GAGAGGGAAATCGTGCGTGACAT	GCTCGTTGCCAATAGTGATGACCT
*ZO-1*	AAGAAGCGATTCAGCAGCAACAGA	AAGGTCATCACTTGTAGCACCATCC
*Occludin*	TGGCTATGGCTATGGCGGATATACA	ACTAAGGAAGCGATGAAGCAGAAGG
*MUC2*	ACCGAGCACATCACCTACCACAT	TCCAGAATCCAGCCAGCCAGTC
*IL-4*	GCCATATCCACGGATGCGACAA	GGTGTTCTTCGTTGCTGTGAGGA
*IL-10*	TCCCTGGGTGAGAAGCTGAAGAC	ACCTGCTCCACTGCCTTGCT
*IL-22*	GTCCAACTTCCAGCAGCCATACAT	GGTAGCACTGATCCTTAGCACTGAC
*IFN-γ*	TCTTGGATATCTGGAGGAACTGGCA	ATGACGCTTATGTTGTTGCTGATGG
*J-chain*	TGTGGAAGTGGAGCTGGAAGATCA	AGGTCTCAGGAACACCATCGTCTT

## Data Availability

The original contributions presented in this study are included in the article or Supplementary Materials. Further inquiries can be directed to the corresponding author.

## Supplementary Materials

The following supporting information can be downloaded at https://www.mdpi.com/article/10.3390/foods14193376/s1. Figure S1. Structural characteristics of GFP. (A) Detection of the average molecular weight. (B) Determination of monosaccharide composition. Figure S2: The relative expression level of (A) pIgR in the colon. pIgR (polymeric immunoglobulin receptor). It serves as a primary defense mechanism against pathogens through the transport of secretory IgA (sIgA) to mucosal surfaces; Figure S3: The classification of differential metabolites in the HMDB hierarchy (Superclass) is displayed: (A) CON vs. MOD. (B) MOD vs. GFP-H.

## Abbreviations

The following abbreviations are used in this manuscript:

GFP	*Grifola frondosa* polysaccharides
CTX	Cyclophosphamide
Ig G	Immunoglobulin G
Ig M	Immunoglobulin M
IFN-γ	Interferon-γ
IL-4	interleukin 4
IL-10	interleukin 10
IL-22	interleukin 22
ZO-1	Zonula occludens–1
MUC-2	Mucin2
SCFAs	Short-chain fatty acids
MAPK	Mitogen-activated protein kinase
DAO	Diamine oxidas
LPS	Lipopolysaccharide
ELISA	Enzyme-linked immunosorbent assay
RT-qPCR	Reverse transcription-quantitative polymerase chain reaction
H&E	Hematoxylin and eosin
AB-PAS	Alcian Blue-Periodic Acid Schiff
sIgA	Secretory IgA
pIgR	Polymeric immunoglobulin receptor
IF	Immunofluorescence
GC-MS	Gas Chromatography–Mass Spectrometer
LC-MS	Liquid Chromatography–Mass Spectrometry
PCoA	Principal co-ordinates analysis
LEfSe	Linear discriminant analysis of effect size
GALT	Gut-associated lymphoid tissue
Th1/Th2	T helper 1 and T helper 2
TJ	Tight junction
