# Supplementary material for: Grifola frondosa Polysaccharides Alleviated Cyclophosphamide—Induced Intestinal Injury Based on Microbiota, Metabolite and Immune Axis Modulation
Source: Foods. 2025 Sep 29;14(19):3376. doi: 10.3390/foods14193376 (PMC12523291; doi:10.3390/foods14193376)
Supplement: Supplementary file 1 [file foods-14-03376-s001.zip › foods-3869212-supplementary.pdf]

Supplementary materials

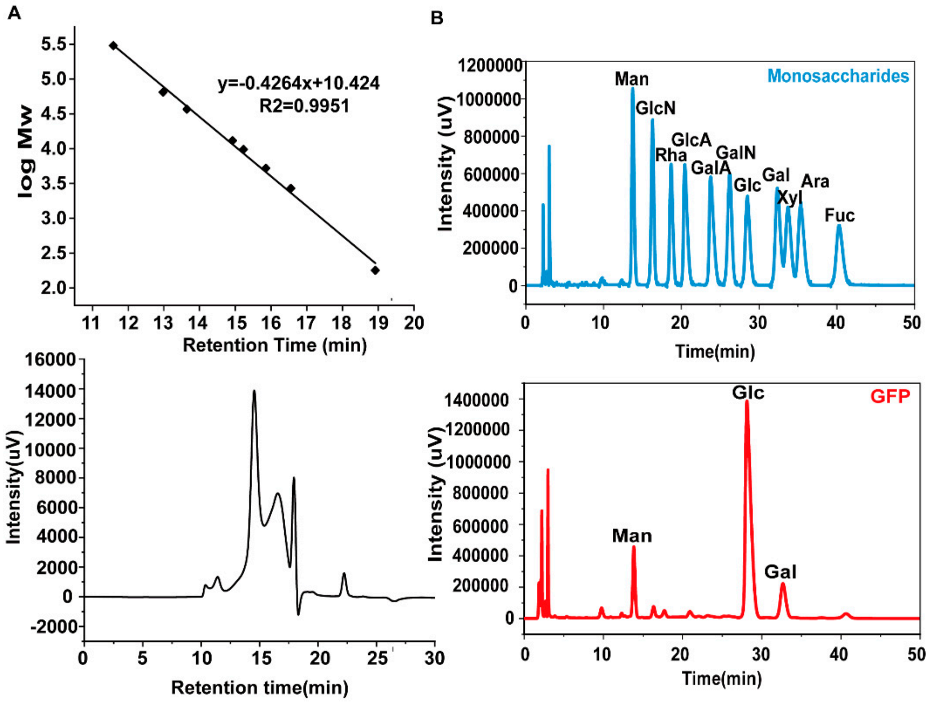

Figure S1. Structural characteristics of GFP. (A) Detection of the average molecular weight. (B) Determination of monosaccharide composition.

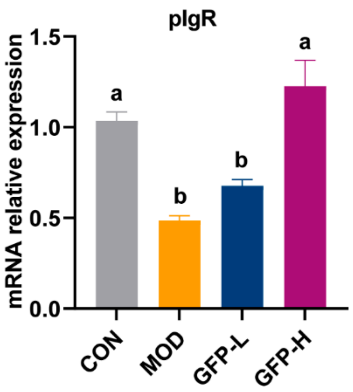

Figure S2. The relative expression level of (A) pIgR in the colon. pIgR(polymeric immunoglobulin receptor). It serves as a primary defense mechanism against pathogens through the transport of secretory IgA (sIgA) to mucosal surfaces.

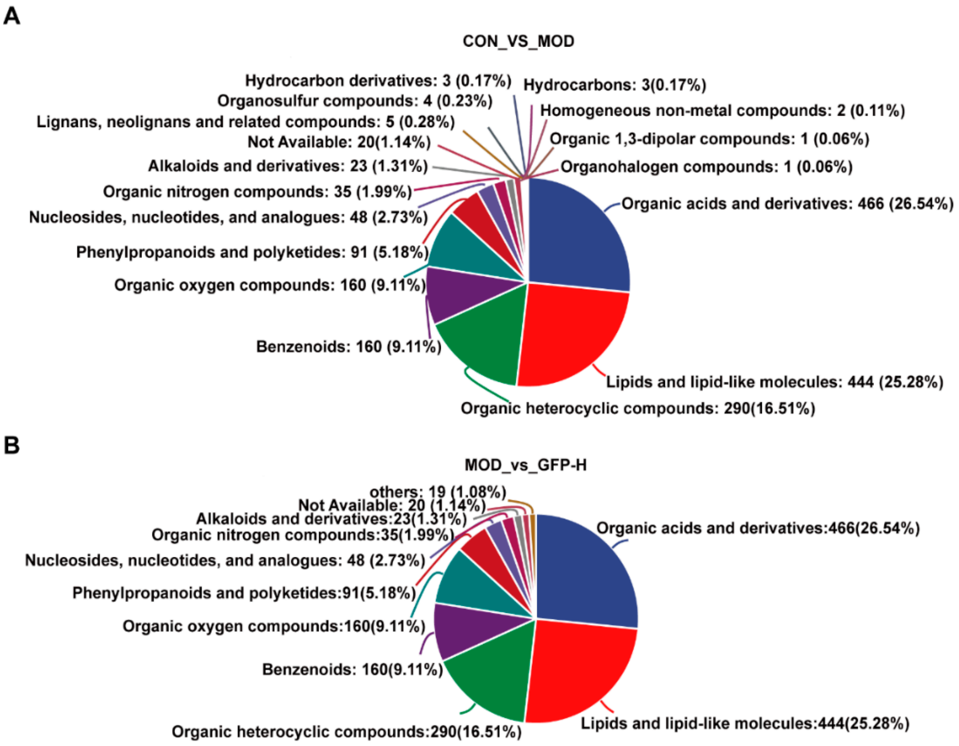

Figure S3. The classification of differential metabolites in the HMDB hierarchy (Superclass) was displayed. (A) CON vs MOD. (B) MOD vs GFP-H.
